# Editorial: Radiomics-based theranostics in cancer precision medicine

**DOI:** 10.3389/fonc.2023.1250079

**Published:** 2023-09-12

**Authors:** Jiansong Ji, Shenghong Ju, Wenli Cai

**Affiliations:** ^1^ Department of Radiology, Lishui Central Hospital, Lishui, China; ^2^ Department of Radiology, Southeast University, Nanjing, China; ^3^ Department of Radiology, Harvard Medical School, Boston, MA, United States

**Keywords:** cancer, radiomics, theranostics, imaging, precision medicine

Cancer is the second-leading cause of death worldwide and represents a large barrier to prolonging life expectancy. Cancer incidence and cancer-related mortality are rising. Lung cancer is the leading cause of cancer-related death, with approximately 1.8 million deaths worldwide (representing 18% of all cancer deaths), and breast cancer is the most commonly diagnosed cancer, with an estimated 2.3 million new cases globally in 2020 (representing 11.7% of cancer cases) (1). The management of cancer includes traditional surgery, precision/minimally invasive surgery, molecular imaging support, and, more recently, robot- or artificial intelligence (AI)-assisted surgical procedures (2). Combination therapy has been widely used to improve survival rates and reduce the side effects of treatment. Over the past few decades, cancer diagnosis and treatment strategies have been revolutionized. Medical imaging plays a pivotal role in the diagnosis and treatment of cancer because it can comprehensively assess the tumor and its environment. A wide variety of imaging modalities are used for theranostics, including optical (fluorescence or bioluminescence), nuclear (positron emission tomography [PET] or single-photon emission computerized tomography [SPECT]), ultrasound, photoacoustic, computed tomography (CT), and magnetic resonance (MR) imaging techniques.

Radiomics is an emerging tool in personalized medicine that mines features of medical images and translates high-throughput imaging features to quantitative data for predictive or prognostic purposes (3). As a bridge between medical imaging and personalized medicine, radiomics is becoming increasingly important in tumor diagnosis, treatment decisions, and prognosis prediction. Therefore, radiomics may provide quantitative and objective support for decisions surrounding cancer detection and treatment. Recently, research efforts have focused on the normalization and verification of radiomics algorithms to demonstrate their usefulness and robustness.

In this Research Topic, we focus on the most recent research on radiomics features extracted from CT, MRI, and ultrasound images to predict cancer biomarkers, treatment effectiveness, cancer progression, and cancer differential diagnoses. Through internal and external validation, one study evaluated the ability of peritumoral, intratumoral, and combined CT radiomics features to predict chemotherapy response in non-small cell lung cancer (NSCLC). The authors concluded that noncontrast CT radiomics features from both the peri- and intratumoral regions could predict the chemotherapy response in NSCLC through machine learning models; furthermore, the 0–3 mm peritumoral region presented better performance than the peri- and intratumoral regions (Chang et al.). An accurate and reproducible model was constructed to predict the response of anti-PD-1 therapy for advanced NSCLC, which demonstrated the robustness of combining radiomics and deep learning features with machine learning methodologies (Ren et al.). A retrospective study analyzed the pretreatment CT images and clinical information of 692 patients with lung adenocarcinomas to predict their epidermal growth factor receptor (EGFR) mutation status and response to first-line tyrosine kinase inhibitors (TKIs) (Jiang et al.). For patients with small cell lung cancer (SCLC), CT-based radiomics integrated with CA125 and CA72-4 provided individualized pretreatment prediction of the response to platinum treatment (Su et al.). An accurate, rapid, and noninvasive indicator is needed to predict the efficacy of anti-angiogenic therapy in patients with advanced colorectal liver metastases (CRLMs); therefore, dynamic radiomics features from different sequences in the same patient were applied to predict treatment efficacy (Qu et al.). Considering the distinct phenotypic and biological characteristics of different pathological subtypes of lung cancer, it is important to differentiate between the pathological subtypes of NSCLC before clinical management. Nomograms combined with clinical parameters and radiomic features from pretherapy dual-energy computed tomography images presented suitable performance in distinguishing between adenocarcinoma (ADC) and squamous cell carcinoma (SCC), with an area under the curve (AUC) of 0.93 in the training set (Chen et al.). To better understand the significance of vascular endothelial growth factor (VEGF) and p53 in patients with spinal giant cell tumor of the bone (GCTB), a multiparametric model based on CT-based radiomics was constructed. The results indicated that p53 and VEGF are associated with poor prognosis in patients with spinal GCTB. Since T2-weighted imaging (T2WI) and the dynamic enhanced portal venous phase (PVP) of MRI can portray the biological characteristics of pancreatic lesions, another study verified the ability of MRI-based radiomics nomograms to evaluate lymph node metastasis (LNM) in patients with pancreatic ductal adenocarcinoma (PDAC) (Shi et al.). Ultrasound is a repeatable, cost-effective, and routinely used modality. A multicenter retrospective analysis developed an ultrasound radiomics-based nomogram to assess the prognosis of patients with nodal diffuse large B-cell lymphoma; the results demonstrated that this tool could be helpful to further individualize therapy (Deng et al.). Ultrasound radiomics has been employed in clinical practice for the management of breast cancer, with applications in lymph node status evaluation, differential diagnosis, cancer staging, neoadjuvant chemotherapy response prediction, and survival prediction (Gu and Jiang).

In conclusion, with the development of state-of-the-art AI techniques, the underlying information of oncology images can be excavated to assist clinical decision-making. This Research Topic discussed and verified the usefulness of radiomics-based prediction models. For example, CT-derived radiomics models have been established to assess treatment response, MRI-based radiomics parameters have been applied to predict lymph node metastasis, and ultrasound-based radiomics can be used to personalize breast cancer management and predict the overall survival of patients with nodal diffuse large B-cell lymphoma. Overall, radiomics features derived from medical images can translate qualitative information to quantitative data, broadening the applicability of medical images in cancer theranostics.

## Author contributions

All authors listed have made a substantial, direct, and intellectual contribution to the work and approved it for publication.
